# Fortune telling addiction: Unfortunately a serious topic. About a case report

**DOI:** 10.1556/JBA.4.2015.1.7

**Published:** 2015-03-18

**Authors:** MARIE GRALL-BRONNEC, SAMUEL BULTEAU, CAROLINE VICTORRI-VIGNEAU, GAËLLE BOUJU, ANNE SAUVAGET

**Affiliations:** ^1^Addictology Department, Nantes University HospitalNantesFrance; ^2^EA 4275 “Biostatistics, Pharmacoepidemiology and Subjective Measures in Health Sciences”, Nantes UniversityNantesFrance; ^3^Psychiatry Department, Nantes University HospitalNantesFrance; ^2^EA 4275 “Biostatistics, Pharmacoepidemiology and Subjective Measures in Health Sciences”, Nantes UniversityNantesFrance; ^4^Pharmacology Department, Nantes University HospitalNantesFrance; ^1^Addictology Department, Nantes University HospitalNantesFrance; ^2^EA 4275 “Biostatistics, Pharmacoepidemiology and Subjective Measures in Health Sciences”, Nantes UniversityNantesFrance; ^3^Psychiatry Department, Nantes University HospitalNantesFrance; ^1^Addictology Department, Nantes University HospitalNantesFrance; ^2^EA 4275 “Biostatistics, Pharmacoepidemiology and Subjective Measures in Health Sciences”, Nantes UniversityNantesFrance; ^3^Psychiatry Department, Nantes University HospitalNantesFrance; ^4^Pharmacology Department, Nantes University HospitalNantesFrance

**Keywords:** behavioral addiction, behavioral problems, clairvoyance, fortune telling

## Abstract

**Background:**

Constant social change brings about new forms of behavior, such as smartphone use, social networking, indoor tanning, cosmetic surgery, etc., that could become excessive or even lead to new forms of addictive disorders.

**Methods:**

We report the case of a woman who starts consulting for “clairvoyance addiction”. We then discuss the addictive nature of her disorder, based on several classifications of addiction.

**Results:**

The patient fulfilled the criteria for addiction and her clinical features were typical of that of addicted people. Other differential diagnoses were discussed.

**Conclusion:**

As for any addictive behavior, the interaction of several risk factors should be considered. They are related to the individual himself, but also to the object of addiction and to the socio-environmental context. In this case, all the conditions were met for fortune telling use to become addictive.

## INTRODUCTION

Addictions cannot be reduced to substance use disorders (SUD). Indeed, it is now widely agreed that they can also be related to non-drug behaviors (e.g. gambling or shopping) and to substances that had not until then been viewed as addictive (e.g. food) (Gearhardt, White & Potenza, 2011). Whether they are linked to SUDs or non-drug behaviors, these addictions actually have a great deal in common despite their apparent clinical heterogeneity. Mainstream thinking on the subject increasingly views addictions as a coherent whole. This can be observed in the latest version of the DSM. The DSM-5 (APA, 2013) now includes a section entitled “Substance-related and addictive disorders”, which combines SUDs and gambling disorders (O’Brien, 2011). Although they are not included in the DSM-5, several other disorders that are linked to the Internet, sex, exercise and shopping (Grant, Potenza, Wienstein & Gorelick, 2010) are considered in this section. It should be expected that this list is non-exhaustive; indeed, constant social change brings about new forms of behavior, such as smartphone use, social networking, indoor tanning, cosmetic surgery, etc., that could lead to new forms of addictive disorder (Andreassen & Pallesen,2013; Harth & Hermes, 2007; Kwon etal., 2013; Petit, Lejoyeux, Reynaud & Karila, 2013). Clairvoyance consulting, also known as fortune teller consulting, is a behavior that may seem harmless, but can also become excessive. Fortune telling is defined as the practice of predicting information about a person’s life, using for example horary astrology, cartomancy or crystallomancy. To date, research on fortune telling has only focused on the sociological or psychological aspects (Hughes, Behanna & Signorella, 2001; Irwin, 1993; Shein, Li & Huang, 2014; Taneda, 2000). To the best of our knowledge, we could not find any study or case report about an addictive use of clairvoyance. However, blogs and discussion forums on the Internet indicate that such a form of addictive disorder could exist, since parallels can be drawn with other more common types of addictions, such as the existence of adverse consequences, and the help some people with a problematic use of fortune telling decide to seek from professionals. For these reasons, we found interesting to report the case of a woman seeking treatment for an excessive use of fortune tellers consulting. Indeed, this case arouses significant diagnostic issues, but also risk factors issues, which are discussed later on in this paper.

## CASE REPORT

Helen is a 45-year-old woman who declares early on suffering from “a clairvoyance addiction”. That is precisely why she consults at our department, which is specialized in the field of addictive disorders. She has no particular medical history, except for two major depression episodes after romantic breakups, and does not take any medication. She regularly sees a psychiatrist for support psychotherapy because of negative life events (sexual abuse and death in her family). She is divorced and does not have any children. Her career as a manager seems to fully satisfy her. She decides to seek treatment on account of her excessive financial expenditures due to the consultation of fortune tellers. Another motivation that explains her decision is her age. Indeed, she says she is entering a new phase in her life, after renouncing to the idea of becoming a mother one day.

Helen first consulted a fortune teller when she was 19. She was then looking for advice on her secondary education orientation. She points out that she has always had difficulties making decisions because she is always afraid to make the wrong choices. She kept a good memory of this consultation, because of the reassuring feeling it gave her. She episodically consulted fortune tellers over the next few years. Between the ages of 25 to 27, however, she intensified the consultations, ending up losing control of her use of fortune telling. She was then in a relationship and was hoping that the fortune tellers could answer her obsessive doubts: *“Does he really love me?” “How long will our relationship last?” *and after their breakup: *“Will he come back?”*

The episode she is currently undergoing dates back to the beginning of her marriage when she was 37. She repeatedly returned to fortune telling to reassure herself about the future of her relationship, and increasingly so as it deteriorated. The breakup worsened the disorder. Since her divorce, she consults fortune tellers– not always the same person – on the phone or online, in a compulsive way, more and more often (up to every day), for longer and longer periods of time (up to 8 hours a day) and spends each time more and more money (up to 200 euros per session). As she is never satisfied with the fortune tellers’ predictions, she will consult again very soon after the latest call or connection. Every choice she has to make, from the most trivial (going to the movies) to the most important (making relationship decisions), leads her to irrationally consult a fortune teller. During the consultation, she is absolutely convinced that the fortune teller can guess the future and that his/her predictions will come true. She says feeling excitement before each consultation, but also nervous tension and anxiety. Consulting fortune tellers relieves her of this psychological discomfort, but only temporarily so, after which she feels guilty. This excessive behavior gives her some kind of reassurance and allows her to make up for her lack of self-confidence. In that sense, the excessive behavior could be considered as an attempt at self-medication or as a way to cope with negative emotions. However, Helen knows that her belief in the fortune tellers’ ability to predict the future is completely irrational. This brings major adverse consequences, particularly in financial terms: despite a comfortable income, she is indebted. She also says having low self-esteem, due to her inability to resist her strong urge to consult fortune tellers, and due to her being isolated from the others because of the time spent consulting fortune tellers. Helen succeeds in limiting the consultation of fortune tellers during short periods of time, when her financial situation becomes too critical. Table 1 provides further information on this behavior.

## DISCUSSION

This case report arises three main issues: its consideration as an addictive-like phenomenon, its differential diagnoses and the individual vulnerability for an addictive behavior.

Considering the first issue, addictions might be defined as “a condition in which a behavior that can function both to produce pleasure and to reduce painful affects is employed in a pattern that is characterized by two key features: (1) recurrent failure to control the behavior (here, fortune tellers’ consultation), and (2) continuation of the behavior despite significant harmful consequences (here, financial losses and social isolation)” (Goodman, 2008). We checked that the disorder met the criteria proposed by Goodman when he described sexual addiction, since those criteria are relevant to all addictions, whether they are behavioral addictions or drug addictions (Goodman, 1990) (see Table 1). The addictive behavior is learned by operant conditioning, with expectation of positive effects (the behavior is then arousal driven: Helen describes that she is sometimes excited before a consultation) and with expectation of the release of negative effects (the behavior is then anxiety-driven and aimed at alleviating anxiety and doubt). More recent concepts could also be used ((Demetrovics & Griffiths, 2012) & Griffiths, 2012). Griffiths conceptualized addiction using a “component model”, with distinct common components (salience, mood modifications, tolerance, withdrawal, conflict and relapse) (Griffiths, 2005). According to the DSM-5, only gambling disorder has sufficient data to be included in the “Substance-related and addictive disorders” section (APA, 2013). But its diagnostic criteria can be applied to the problematic fortune telling use, with minor adjustments (see Table 1).

**Table 1.** Relevant models of addiction applied to the case report

DSM-5 model (based on the Gambling Disorder diagnostic criteria)				
A. Persistent and recurrent gambling behavior leading to clinically significant impairment or distress, as indicated by the individual exhibiting four (or more) of the following in a 12-month period:				
1.	Needs to gamble with increasing amounts of money in order to achieve the desired excitement		√	
2.	Is restless or irritable when attempting to cut down or stop gambling.		√	
3.	Has made repeated unsuccessful efforts to control, cut back, or stop gambling.		√	
4.	Is often preoccupied with gambling.		√	
5.	Often gambles when feeling distressed.		√	
6.	After losing money gambling, often returns another day to get even.		√	
7.	Lies to conceal the extent of involving with gambling.		√	
8.	Has jeopardized or lost a significant relationship, job, or educational or career opportunity because of gambling.		Not fulfilled	
9.	Relies on others to provide money to relieve desperate financial situations caused by gambling.		√	

B. The gambling behavior is not better explained by a manic episode				
Griffith’s “components” model				
Salience		Consulting fortune tellers becomes the most important activity in Helen’s life and dominates her thinking (preoccupation and cognitive distorsions), feelings (cravings) and behavior (she has progressively quit all her leisure activities, particularly going out with friends).		
Mood modification		Helen says feeling excitement before each consultation, but also feels nervous tension and anxiety. This excessive behavior gives her some kind of reassurance and the excessive behavior could be considered as an attempt at self-medication or a way to cope with negative emotions.		
Tolerance		Over time, Helen has been feeling a growing need to consult fortune tellers, and the consultations have to last longer to obtain the same effect of relief.		
Withdrawal		When she attempts to resist the urge to consult or has to refrain from consulting fortune tellers (in the case of her financial situation being too critical, for example), she feels tense and nervous.		
Conflict		Helen knows that her use of fortune telling is problematic, and that it brings very negative consequences. However, she cannot refrain from consulting fortune tellers, leading to an intra-psychic conflict and guilt.		
Relapse		Over the years, Helen has made repeated efforts to reduce and stop this problematic behavior. Her clinical course is characterized by relapses and remissions.		

Goodman’s model				
A	Recurrent failure to resist impulses to engage in a specified behavior.			√
B	Increasing sense of tension immediately prior to initiating the behavior.			√
C	Pleasure or relief at the time of engaging in the behavior.			√
D	A feeling of lack of control while engaging in the behavior.			√
E	At least five of the following:			
	E1. Frequent preoccupation with the behavior or with activity that is reparatory to the behavior.			√
	E2. Frequent engaging in the behavior to a greater extent or over a longer period than intended.			√
	E3. Repeated efforts to reduce, control or stop the behavior.			√
	E4. A great deal of time spent in activities necessary for the behavior, engaging in the behavior or recovering from its effects.			√
	E5. Frequent engaging in the behavior when expected to fulfill occupational, academic, domestic or social obligations.			√
	E6. Important social, occupational or recreational activities given up or reduced because of the behavior.			√
	E7. Continuation of the behavior despite knowledge of having a persistent or recurrent social, financial, psychological or physical problem that is caused or exacerbated by the behavior.			√
	E8. Tolerance: need to increase the intensity or frequency of the behavior in order to achieve the desired effect or diminished effect with continued behavior of the same intensity.			√
	E9. Restlessness or irritability if unable to engage in the behavior.			√
F.	Some symptoms of the disturbance must have persisted for at least 1 month or have occurred repeatedly over a longer period.			√

Regarding the second issue, the only few differential diagnoses that would better explain the described clinical situation are an obsessive compulsive disorder (OCD), a generalized anxiety disorder (GAD) and an unspecified anxiety disorder (UAD). As regards the OCD, Helen is indeed an undecided woman: she obsesses over her doubts about relationships and more generally, about every choice she has to make. She makes irrational decisions when she consults fortune tellers, highlighting her poor decision making abilities. Moreover, she unsuccessfully attempts to resist her craving to consult fortune tellers and acts in a compulsive way when she phones them for a consultation. The effect of consulting a fortune teller seems to be a transient doubt alleviation more than the feeling of escape we can observe in addictive behaviors. However, she does not meet the diagnosis criteria for OCD, according to the DSM-5 (APA, 2013) (see Table 2). Her doubts and preoccupations with her behavior do not belong to the conventional obsessive thoughts (such as contamination obsessions for instance) and she does not find these ideas difficult to resist. What she does find difficult to resist is the urge to consult fortune tellers. The ego-syntonic nature of her behavior contrasts with the usual ego-dystonic nature of OCD – although the ego-dystonic nature of OCD is not always present (Grant et al., 2010). Consulting fortune tellers, even in a compulsive way, does not make her behavior stereotyped, since she does not have a chosen way of having these consultations: it can be either by phone or on the Internet. Moreover, Helen wishes to resist her compulsive behavior only because of its deleterious consequences. Lastly, a clinical course composed of periods of relative control or abstinence alternating with periods of relapse can be observed here. On the other hand, the natural course of OCD usually consists in a progressive worsening. However, some authors consider that the obsessive compulsive spectrum includes some addictive behaviors, such as pathological gambling (Hollander, 1993). We can also evoke a GAD, which is defined as an excessive anxiety state about a variety of events and situations. However, this disorder can be easily eliminated because Helen does not think things will always go badly. She simply worries about the decisions she has to make. Moreover, she does not exhibit symptoms such as irritability, muscular tension or restlessness. Finally, Helen could indeed suffer from an UAD insofar as her primary symptom appears to be anxiety. However, the main diagnosis criteria for each specified anxiety disorder is lacking.

**Table 2.** Diagnostic criteria for Obsessive-Compulsive Disorder (DSM-5) applied to the case report

A	Presence of obsessions, compulsions, or both		

	*Obsessions are defined by (1) and (2):*		
	1.	Recurrent and persistent thoughts, urges, or images that are experienced, at some time during the disturbance, as intrusive and unwanted, and that in most individuals cause marked anxiety or distress.	Helen experiences obsessive doubts about the future of her relationship, but also about the choices she has to make in her daily life. It would be an exaggeration to talk of obsessions, because her thoughts are not recurrent and persistent, but change over time. Moreover, Helen doesn’t consider these thoughts as intrusive and they don’t belong to the conventional obsessive thoughts. She attempts to respond to her doubts by consulting fortune tellers.
	2.	The individual attempts to ignore or suppress such thoughts, urges, or images, or to neutralize them with some other thought or action.	

	*Compulsions are defined by (1) and (2):*		
	1.	Repetitive behaviors or mental acts that the individual feels driven to perform in response to an obsession or according to rules that must be applied rigidly.	Consulting fortune tellers is described as an acting-out, resulting from a loss of control. Helen criticizes her use of fortune telling. She attempts to quit but fails to resist. Consulting fortune tellers is not designed to neutralize her obsessive doubts. Consulting fortune tellers, even in a compulsive way, does not make her behavior stereotyped, since she does not have a chosen way of having these consultations: it can be either by phone or on the Internet.
	2.	The behaviors or mental acts are aimed at preventing or reducing anxiety or distress, or preventing some dreaded event or situation; however, these behaviors or mental acts are not connected in a realistic way with what they are designed to neutralize or prevent, or are clearly excessive.	

B	The obsessions or compulsions are time-consuming or cause clinically significant distress or impairment in social, occupational, or other important areas of functioning.		Her obsessive doubts and compulsive fortune telling consultations are time-consuming and cause clinically significant anxiety. During the worst periods, most of Helen’s free time is spent on fortune telling, to the detriment of domestic or leisure activities.

C	The obsessive-compulsive symptoms are not attributable to the physiological effects of a substance or another medical condition.		Helen doesn’t use any substance or doesn’t have any medical history that could explain her symptoms.

D	The disturbance is not better explained by the symptoms of another mental disorder.		The case report is better explained by symptoms of an addictive-like phenomenon.

Besides, although it may be tempting to mention the presence of psychotic symptoms (because of her irrational, unusual thoughts), we would be inclined to favor cognitive distortions focused on a very specific field of her life. In order to adopt a systematic and rigorous approach, we have associated our clinical assessment to a structured diagnostic interview. According to the results of the MINI (Lecrubier et al., 1997), the main current psychiatric diagnoses (mood disorders, anxiety disorders, psychotic syndrome) were eliminated.

Considering the third issue, Helen’s clinical features are typical of that of addicted people Goodman, 2008, whose personality is characterized by impulsivity (which particularly comes out in cases of an intense emotional context), and by sensation seeking. Other pathological personality traits can be pointed out: emotional lability, separation insecurity and low self-esteem. Moreover, Helen exhibits a decision making profile that is characterized by poor decision making abilities and failure to make the right and rational choices. This profile is suggestive of a predominantly executive dysfunction and is very frequent in addictive disorders (Ahn et al., 2014; Zois et al., 2014), even if it is not specific since other disorders involve decision making deficits, such as OCD (Kashyap, Kumar, Kandavel & Reddy, 2013), Attention Deficit/Hyperactivity Disorder (Hauser et al., 2014) and schizophrenia (Orellana & Slachevsky, 2013). In addition, her life is punctuated by traumatic events, with a history of major depression episodes. Her addictive behavior was onset during young adulthood and her clinical course is characterized by relapses and remissions (Grant et al., 2010).

## CONCLUSION

As for any addictive behavior, the interaction of several risk factors should be considered. These risk factors are related to the individual himself, but also to the object of addiction and to the socio-environmental context. Individual risk factors were described above. Regarding the risk factors related to the object of addiction (i.e. fortune telling use), one might mention, inter alia, the possibility to consult online, which guarantees anonymity. Furthermore, the Internet increases both accessibility and availability. Finally, the money spent during fortune telling sessions seems virtual, which makes it all the more easy to spend. Increased risks related to the Internet have already been described on gambling (Griffiths, Wardle, Orford, Sproston & Erens, 2009). Regarding socio-environmental risk factors, today’s society encourages the need for control and does not give way to uncertainty. In Helen’s case, all the conditions were met for the fortune telling use to become excessive, and we are tempted to conclude that it is an addictive-like phenomenon. Future investigations are needed to confirm the relevance of this disorder and to estimate its prevalence.

